# Indirect Structural Muscle Injuries of Lower Limb: Rehabilitation and Therapeutic Exercise

**DOI:** 10.3390/jfmk6030075

**Published:** 2021-09-13

**Authors:** Stefano Palermi, Bruno Massa, Marco Vecchiato, Fiore Mazza, Paolo De Blasiis, Alfonso Maria Romano, Mariano Giuseppe Di Salvatore, Elisabetta Della Valle, Domiziano Tarantino, Carlo Ruosi, Felice Sirico

**Affiliations:** 1 Department of Public Health, University of Naples “Federico II”, 80131 Naples, Italy; stefanopalermi8@gmail.com (S.P.); b.massa91@gmail.com (B.M.); elisabetta.dellavalle@unina.it (E.D.V.); domiziano22@gmail.com (D.T.); carlo.ruosi@unina.it (C.R.); 2 Sport and Exercise Medicine Division, Department of Medicine, University of Padova, 35128 Padova, Italy; marcovecchiato.md@gmail.com; 3 Rehabilitation Center “Phorma”, 84047 Salerno, Italy; fioremazza@hotmail.it; 4 Section of Human Anatomy, Department of Mental and Physical Health and Preventive Medicine, University of Campania Luigi Vanvitelli, 80131 Naples, Italy; paolodeblasiis@gmail.com; 5 Orthopedics and Sport Medicine Unit, Campolongo Hospital, 84121 Salerno, Italy; alfonso.maria.romano@gmail.com; 6 AORN S. Giuseppe Moscati, UOC Ortopedia e Traumatologia, 83100 Avellino, Italy; mariano.disalvatore@gmail.com

**Keywords:** muscle injury, rehabilitation, sport medicine

## Abstract

Muscle injuries are the most common trauma in team and individual sports. The muscles most frequently affected are those of the lower limb, and in particular hamstrings, adductors, rectus femoris and calf muscles. Although several scientific studies have tried to propose different rehabilitation protocols, still too often the real rehabilitation process is not based on scientific knowledge, especially in non-elite athletes. Moreover, the growing use of physical and instrumental therapies has made it increasingly difficult to understand what can be truly effective. Therefore, the aim of the present paper is to review proposed therapeutic algorithms for muscle injuries, proposing a concise and practical summary. Following a three-phase rehabilitation protocol, this review aims to describe the conservative treatment of indirect structural muscle injuries, which are the more routinely found and more challenging type. For each phase, until return to training and return to sport are completed, the functional goal, the most appropriate practitioner, and the best possible treatment according to current evidence are expressed. Finally, the last section is focused on the specific exercise rehabilitation for the four main muscle groups with a structured explanatory timetable.

## 1. Introduction

Muscle injuries (MI) are the most common trauma in team and individual sports and are responsible for most of the time lost in both training and competition [1,2,3]. In soccer, they account for half of the injuries recorded [1]. In particular, four muscles groups are frequently involved [4,5]: hamstrings are the muscle groups most prone to injury [6,7,8], followed by adductors, rectus femoris and calf muscles.

The chance to receive an accurate early diagnosis and to have proper rehabilitation is different depending on whether these are professional or amateur athletes [9].

It is a common opinion that rehabilitation protocols designed for muscle injuries should be built mostly on available structure and therapeutic options [10], rather than on scientific knowledge. Clinical experience in the treatment of muscle injuries has proven that a wait-and-see approach is not effective [11]. Several therapeutic options for muscular injuries exist and, even if widely used, many reviews could not find enough evidence for conclusion about any of them. Therefore, unfortunately, common opinion has become to consider that what is popular is what is really effective [9,12,13].

Even though several scientific studies have tried to propose different rehabilitation protocols, to design a particular rehabilitation pathway for each muscular injury based on its grade and/or location is a difficult task; moreover, several commercial physical or instrumental therapies are becoming increasingly used for muscle injury treatment and rehabilitation, even if scientific evidence about their use are discordant [9,14].

In subjects affected by muscle injuries, the diagnostic and rehabilitative approach relies on several factors, such as age, gender, athletic demands, muscular groups involved and type of injury. To this scope, several classifications have been proposed over the years. All these classifications are based on some common criteria such as mechanism of injury (direct or indirect) and degree of lesion of muscle tissue (structural or non structural).

In a real-life scenario, the indirect structural muscle injuries represent the most common type of muscle injuries, and some muscle groups of lower limb are affected predominantly.

Therefore, the aim of the present paper is to review proposed therapeutic algorithms for indirect structural muscle injuries of the lower limb, with a particular insight on hamstrings, adductors, rectus femoris and calf muscles rehabilitation.

## 2. Muscle Injuries Rehabilitation

There are several classifications of muscle injuries, such as the Munich Muscle Injury Classification, the ISMuLT (Italian Society of Muscles, Ligaments and Tendons) classification, and the British Athletic Classification, that, if used extensively, could improve diagnosis, prognosis and management of muscle injuries [15]. Depending on the mechanism of trauma, according to ISMuLT classification [9], muscle injuries may be distinguished as direct and indirect; indirect ones are in turn classified as non-structural and structural. While direct muscle injuries are often the result of external forces, indirect muscle injuries are stretch-induced injuries caused by a sudden forced lengthening over the viscoelastic limits of muscles occurring during a powerful contraction [9,15]. Indirect structural muscle injuries (commonly referred as “muscle tears”) are the more commonly found in everyday clinical practice and represent the biggest challenge in rehabilitation, since these lack a precise therapeutic strategy. Structural muscle injuries classification is shown in Table 1.

Severity [16], site [17], tissue [3], and relapse [18] are important features to consider when a muscle injury has been diagnosed. Proximal hamstring and quadricep lesions have a worse prognosis, as well as distal calf injuries; moreover, myotendinous junction lesions seem to have a longer recovery period [19]. Therefore, type and location of muscular injuries can influence recovery strategies [3] and proposed exercises should respect the principles of specificity, progression, and individualization, respecting painful symptomatology [14,20,21]. Moreover, location of injury, properly marked, could be useful for a focused therapy. Minor or moderate partial lesions (3A and 3B) are prevalent in sport rehabilitation and their conservative management is more controversial, since (sub)total lesions (4) are generally intended for surgery.

The muscle tissue repair process is completed in a period depending on the severity of the lesion. During this period, different well-defined biological phases are involved (destruction phase, repair/regeneration phase and remodeling phase) [14,22]. Each of these phases must be characterized by a well-defined type of muscular contraction that is consistent with the biological condition observed within the injured area [14,22].

Although rehabilitation is subdivided into a defined number of steps, the duration of each is different, and progression is not time based, but clinical, functional, and imaging criteria based [10,23]. Therefore, the duration of each phase is consistent with the dynamics of the healing processes occurring in the muscle tissue and with the severity of the injury. Each step of this process has a customized duration in accordance with the clinical and imaging criteria required for proceeding from one phase to the next.

Ultrasonography (US) offers dynamic muscle assessment and is fast and relatively inexpensive, allowing serial evaluation of the healing process [9]. However, it should be noted that ultrasonography of skeletal muscles requires a high level of skill on the part of the sports physician. It is recommended to use a 7.5- to 10.0-MHz transducer, starting with a transversal section. A complete scan through the muscle should be performed for the purposes of anatomical orientation. Any apparent abnormalities should be compared with the contralateral side. The transducer pressure should be as light as possible, since compressing the muscle may obscure smaller injuries. The longitudinal section is added in locations where a disturbance of the muscle structure or a gap is suspected. In addition, the use of novel US technique could help in this difficult diagnostic process, such as echo intensity [24]. When clinical and ultrasonography evaluation are discordant, or for muscles not accessible to US examination, in elite athletes, Magnetic Resonance Imaging (MRI) may be required to confirm or exclude minor structural injuries, since this technique is often used as a second-line investigation in musculoskeletal diseases [25,26]. MRI plays only a marginal role in the follow-up and monitoring of structural injuries because the images do not correlate well enough with the clinical evaluation, causing a potential late return to play (RTP) for the athlete.

A physician is responsible for the diagnosis, for overseeing the entire rehabilitation process, and for clinical and ultrasound monitoring; other professionals (physiotherapists, athletic trainers and coaches) control the correct execution of the rehabilitation program, each one for what they are entitled for [9].

Below, we proposed the three-phase rehabilitation protocol, based on ISMuLT [9] and Italian consensus conference [10] recommendations.

**0 PHASE (0–72 h post injury)**

- Muscle ultrasound allows to detect the structural damage of the skeletal muscle after 36–48 h from injury, because the hemorrhagic collection is maximized after 24 h and decreases after 48 h [9].
- In the immediate post injury period (24–72 h) it is advisable to apply the PRICE (Protection, Rest, Ice, Compression, Elevation) principle [27]. It is widely used, although there are no high quality randomized clinical trials to prove its effectiveness [28,29,30]. In clinical practice, immediate compression with 15 min cryotherapy cycles, with ice-free phases between, is recommended. Compressive cryotherapy (CC) [31], namely the association between cryotherapy and the application of pressure, deserves separate consideration: CC duration should be 15–20 min, repeated at intervals of 30–60 min for a total of 6 h, so as to substantially limit both the hemorrhage and the myofibril necrosis at the site of injury [32]. It is advisable to apply a compressive bandage and/or compressive cryotherapy within the range of 40–50 mmHg [23].
- A short rest period and/or relative immobilization immediately after the injury is recommended. This rest period optimizes the formation of connective tissue by fibroblasts, thereby reducing the risk of recurrences. Usually crutches are not necessary, while taping can be useful both for immobilization and liquid drainage. However, rest and immobilization should be reduced to only the first postlesion days (3–5 days) [10,14,22,28]. It would be better to have a short immobilization period followed by a progressive load able to favor the correct progression of healing process (POLICE: Protection, Optimal Loading, Ice, Compression, Elevation) [9].
- In the first 72 h postlesion, physical therapies that induce endothermic processes should be avoided for the possible increase in blood extravasation [3,10,33,34].
- After the first 24 h postlesion, it is a good idea to start performing complete lymphatic draining massages and to replace the compression bandage with an elastic bandage [9].
- After the first 24 h postlesion, there is little evidence about the usefulness of pulsed ultrasound therapy (UST) (1 W/cm^2^) [13] (often used as cryo-ultrasound, with the adjunct of ice therapy) and low-level laser therapy (LT) (500 mW/cm^2^) [9,35,36,37].

**1st PHASE**

- Functional goals [9]: treatment of predisposing factors and antagonist muscles; pain-free activity of daily life; pain-free strength training of the injured muscle, at least 50% of theoretical maximum load; recovery of at least 90% of the extensibility deficit of the injured muscle.
- Figure: physician and physiotherapist.
- Location: gym.
- Red Flags [9]: presence of pain when performing strength exercises or low-speed running on the treadmill.
- Image criteria: US check on the 2nd and 4th−5th day after injury [9].

At the beginning of the first phase (second postlesion day), the necrotized parts of the muscle fibers are removed by the macrophages, with an inflammatory process [9]. At the same time, the formation of the scarring connective tissue within the central lesion zone by fibroblasts starts [14,38,39]. Considering that the first 5–7 postlesion days are characterized by a not sufficiently dense and compact scarring, the major risk in this period is that an excessive muscle contraction increases the already existing lesion gap.

- The type of contraction recommended in this first phase is an isometric modality. In fact, during the isometric contraction, there is no myofilaments slippage and, therefore, there is no macro change of the muscle length [22,40]. Between 30 and 50 repetitions of 10–20 s of contraction under the threshold of pain are suggested. According to biomechanics concepts, the internal torque varies along the range of movement (ROM) of each joint. Each joint has specific degrees within the ROM in which the muscle is able to generate the maximum internal force and the anatomical position of muscle–tendon–bone unit give a maximum internal moment arm, generating the maximum torque. To gradually increase mechanical stress on the damaged muscle, it is necessary to proceed along the ROM gradually, by proposing contraction in ROM position where internal force is not able to produce the highest tension of the muscle.
- It is important to correctly perform exercises to recover the extensibility of injured muscle (passive, assisted/active, static or dynamic) [9], and better if with functional schemes. All exercises must be under the threshold of pain. An increased joint range was verified for stretches performed following functional patterns. In case of bi-articular muscles, it is advisable to stretch both insertional areas [41,42].
- Deep massages on the affected area should be avoided [10].
- Elastic bandage is continued until there is liquid collection.
- If there is an excessive hematoma formation within the injured area, it is advisable to proceed to an echo-guided aspiration before the hematoma organization [43].
- It is useful to start an aerobic workout as soon as possible, using non-injured muscles (i.e., upper trunk aerobic workout) [9].
- At the end of each working session, ice massage should be performed for 15–20 min [9].
- The use of electrical stimulation should be encouraged from the first postlesion days to the end of the regeneration phase (up to about the third postlesion week) [10,44,45,46]. Transcutaneous electric nerve stimulation (TENS) is the form of electrical stimulation most recommended in its two forms: conventional and acupuncture-like; several trials highlight its potential role in inhibition of transmission of pain signals [44]. Neuromuscular electrostimulation (NMES) utilizes high-intensity electrical stimulation to elicit intermittent contraction and relaxation of proximal muscle fibers; it is widely prescribed for physical rehabilitation and muscle strengthening [44]. It has been demonstrated that these two techniques can stimulate the implantation of muscle resident stem cells inside the injured area, along with the voluntary exercise performed during rehabilitation [47,48,49].
- There is limited evidence that UST is able both to increase the levels of basic growth factors and to have an antalgic effect [50,51]: it may be recommended after the 0 phase (2 W/cm^2^, in continuous modality, 1 MHz) [10].
- Many studies have shown that LT can reduce the inflammatory process of the damaged muscle tissue [52], speed up the tissue regeneration [53], optimize the oxidative metabolism [54] and stimulate cell proliferation [55,56]. Therefore, the use of LT appears to be justified by sufficient evidence, even if not high quality featured [9,10,57].
- Hyperthermia therapy (HT) has proven to be able to stimulate the tissue repair processes, diminish pain symptoms, increase tissue flexibility, and reduce muscular and joint stiffness [58,59,60,61,62,63,64,65,66]. However, there are poor specific evidence on the HT effectiveness in muscular injuries [9,10].
- Analgesic (paracetamol) can be used in case of pain in the first postlesion days [9,10,67,68], while muscle relaxants, mesenchymal stem cells (MSCs) and platelet-rich plasma (PRP) injections require further evidence-based studies to evaluate their effectiveness [23,69]. The use of nonsteroidal anti-inflammatory drugs (NSAIDs) is controversial [70], and it is not recommended.

**2nd PHASE**

- Functional goals [9]: absence of pain or feeling of diversity in injured muscle when performing exercises; complete recovery of the extensibility of the injured muscle; recovery of the aerobic sport-specific parameters; complete recovery of the pre-injury weight.
- Figure: physiotherapist and athletic trainer.
- Location: gym and sport-field.
- Clinic criteria [10,23,71]: resolution of swelling, if initially present; absence of pain in response to maximal isometric contraction; absence of pain in response to end-range stretching tests carried out in the active and passive modes; complete range of motion (ROM) of the joints involved in the movement.
- Imaging criteria [10,72,73]: resolution of the lesion gap as observed with US or MRI imaging; the presence of granulation repair tissue within the cicatrix zone (CZ) as revealed by the US. US findings observed during normal healing depend on the nature of the original injury and initial sonographic findings. Minor lesions may increase in echogenicity during the healing process. In these cases, a progressive reduction in intensity or its disappearance is considered normal. More prominent lesions may present as hypoechogenic regions with adjacent fluid collection. Resolution or substantial decrease in the quantity of fluid is to be expected during the normal healing process [74,75].
- Red Flags [9]: extensibility test still positive.

At this stage, the scar area in the CZ is further condensed and reduced in size, and myofibers fill the residual gap of the CZ [14,38,76,77]. During this phase, the granulation tissue gains compactness and elasticity [78]. In this regenerative phase mechanical stimuli should be performed in order to induce an optimal tissue repairing [9].

- There is the introduction of progressively intense concentric exercises. During a concentric contraction, the bulk of the muscle shortens due to the sliding motion of the myofilaments with a relatively constant force proportional to the external load, so the CZ is not subjected to traction and the jagged muscle edges, avoiding diastasis [79]. The concentric contraction should be slow and controlled; they can be manual at the beginning, and subsequently with isotonic equipment [80]. Sixty percent of one repetition maximum (RM) should not be exceeded when performing these exercises in this stage [79,80]. The eccentric phase of the movement must, in all cases, be reduced to the minimum possible intensity [10].
- Keep performing exercises to recover the extensibility of injured muscle [9].
- Proprioceptive exercises should be started [9,81]: balance exercises on stable or unstable different shape surfaces, with or without recurrent destabilization, with or without request for additional cognitive tasks, if possible, with the support of the visive system.
- The practice of massage can be introduced as the completion of tissue healing processes has started [10].
- A ‘core stability program’ should be introduced in the rehabilitation plan [10,82,83], eventually combined with proprioceptive exercises [9].
- Aerobic exercises can be introduced during this phase [10,23]: the time-progression should be stationary bike, elliptical machine, anti-gravity running and, finally, treadmill running.
- Physical therapies started could be continued in this phase.

**3rd PHASE**

- Functional goals [9]: consolidation of the strength and extensibility characteristics of the injured muscle; recovery of the sport-specific skills; recovery of the high-intensity sport-specific athletic parameters; working resistance of the injured muscle.
- Figure: physiotherapist and athletic trainer.
- Location: gym and sport-field.
- Clinic criteria [10,23,71]: absence of pain in response to concentric contraction performed at increasing intensity against resistance; absence of pain in response to submaximal eccentric contraction.
- Imaging criteria [10,72,73]: substantial disappearance of the lesion gap on US or MRI examination; presence of compact granulation repair tissue as revealed by US or MRI. Over time small tears may fill with echogenic material, likely representing scar tissue visible at US [84,85]. More extensive scarring results in increased likelihood of recurrent injury [25].
- Red Flags [9]: “different” muscle feeling during or after training.

In this phase, the myofibers intertwining is effectively completed by the interposition of a small amount of scar tissue. There should be proposed strength and extensibility exercises that induce remodeling of the repair tissue based on the sport played [9], depending also on the movement that caused the injury. The remodeling phase may last more than 60 days, depending on the anatomical extent of the injury [9].

- Exercises predominantly based on eccentric contractions of progressively increasing intensity [9,10,23,86,87,88] could be started after an effective concentric contraction is reached. These should be muscle and location specified [89]. These can be performed even with the use of elastic resistance bandages, where the intensity of the eccentric phase is progressively increased [10,23]. Even if some authors suggest introducing eccentric exercises as soon as possible in the rehabilitation protocol [9], the 3rd phase should be the preferred one for their execution. Moreover, evidence about isoinertial exercises are increasing [90].
- There could be the inclusion of isokinetic exercises [10,28].
- Stretching must be introduced gradually and exercises must not cause the onset of pain. The time of elongation initially is 10–15 s and subsequently up to 1 min, in order to induce a durable, and not just a transient, plastic deformation within the area of structural reorganization [10,23]. For bi-articular muscles, please consider both origin and insertion tendons.
- Running could be improved during this phase, on the condition that dynamometric values of the injured muscle have been reinstated to at least 70% of the preinjury level or that of the opposite limb [10,91], and with the use of GPS monitoring [9].
- Sport-specific exercises can be introduced with caution at the end of the third phase [9,10].
- Even if not supported by strong scientific evidence, physical therapies can be used to avoid muscular fatigue, complications, and re-injury [9]: LT [92], ice water immersion [93], contrast therapy [94], HT [95], TENS [96] and extra-corporeal shock wave therapy (ESWT).

It is important to consider an athlete as “healed” as long as three concepts are respected [9]: progression in the recovery of match intensity; continuous information exchange between coaches, trainers, physiotherapist, athlete and physician; and continuous monitoring of the injured muscle characteristics after trainings and matches.

## 3. Specific Exercise Rehabilitation

Even though there are so many rehabilitation exercises used, it is the authors’ opinion that each muscle injury should be treated differently, trying to individualize it as much as possible. They should follow a well-structured timetable that is appropriate for the specific injury or disorder: as we stated before, the correct progression should be isometric (1st phase), concentric (2nd phase) and eccentric (3rd phase) exercises; proprioceptive, neuromuscular and stretching exercises also have a major role in the rehabilitation process. Below we propose examples of exercises for hamstrings (Table 2), rectus femoris (Table 3), adductors (Table 4), and calf injuries (Table 5), along with their criteria for RTT and secondary prevention programs.

**Hamstring**

**Table 2:** Hamstring rehabilitation exercises.

Name	Image	Reference
**Isometric exercises**		
(In case of proximal hamstring lesion)	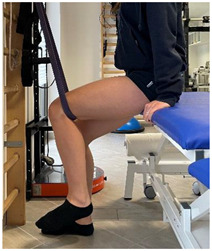	[23]
(In case of medial or distal hamstring lesion)	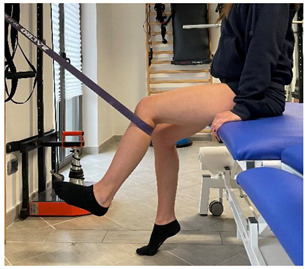	[23]
Isometric exercise at different angles	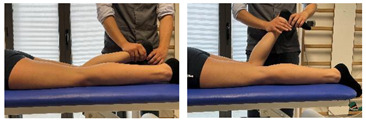	[23]
**Dynamic exercises**		
The extender	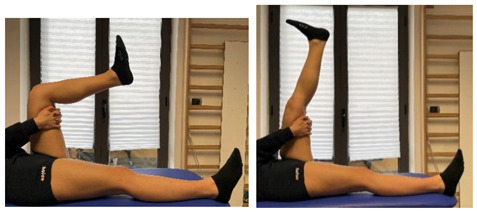	[88,97]
The glider	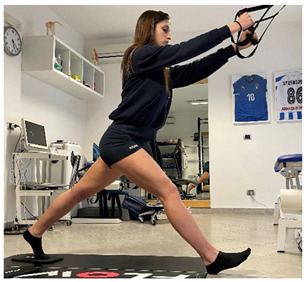	[88,97]
Nordic hamstrings	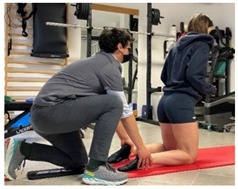 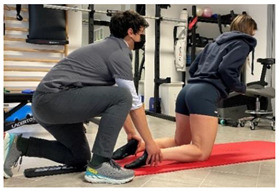	[97]
**Proprioceptive, neuromuscular and stretching exercises**		
Pendulum	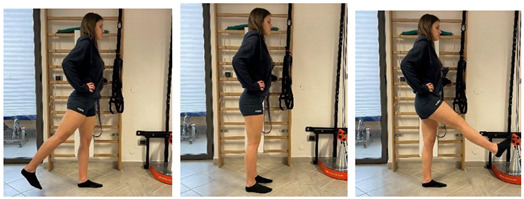	[97]
Stretching Single Leg Raises	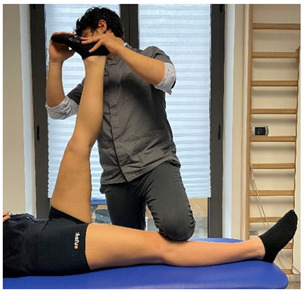	[97]
**Secondary prevention exercises**		
Eccentric knee flexor stretch	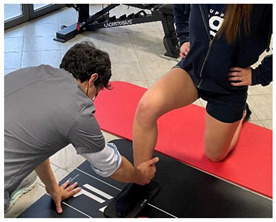 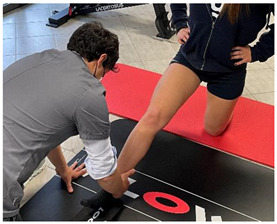	[98]
Eccentric hip extensor stretch	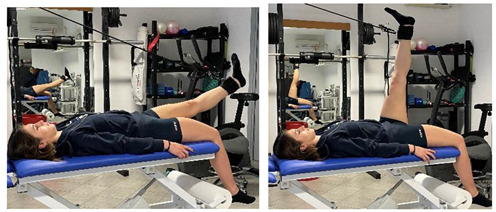	[98]

**Quadriceps**

**Table 3:** Quadriceps rehabilitation exercises.

Name	Image	Reference
**Isometric exercises**		
(In case of proximal lesion)	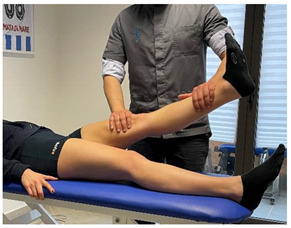	[23]
(In case of medial or distal lesion)	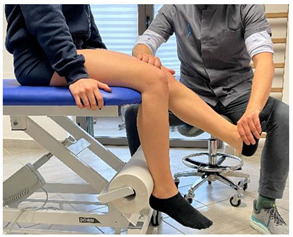	[23]
**Dynamic exercises**		
(In case of proximal lesion)	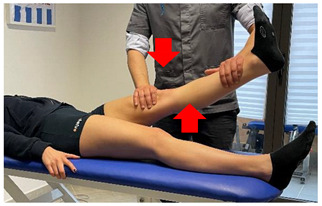	[23]
(In case of medial or distal lesion)	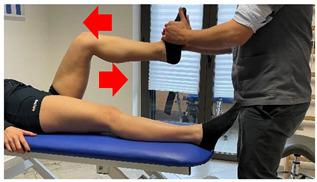	[23]
**Secondary prevention exercises**		
Eccentric hip flexor and knee extensor stretch (eccentric load to rectus femoris)	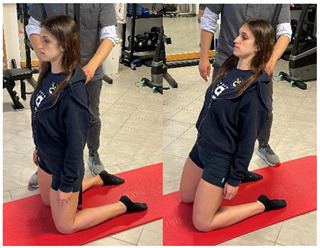	[98]

**Adductors**

**Table 4:** Adductors rehabilitation exercises.

Name	Image	Reference
**Isometric exercises**		
Isometric exercise with ball	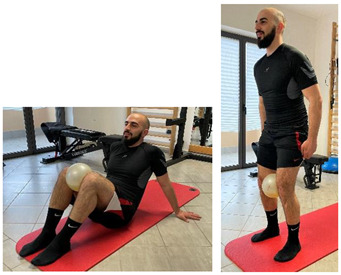 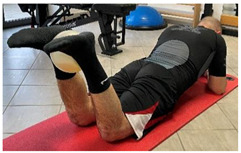	[23]
**Dynamic exercises**		
Manual resisted adduction	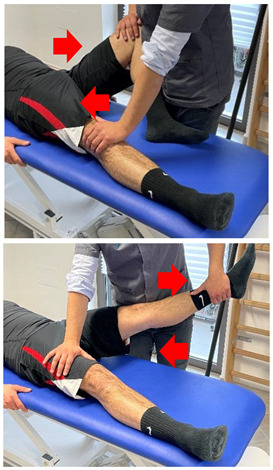	[23]
Adduction with elastic resistance	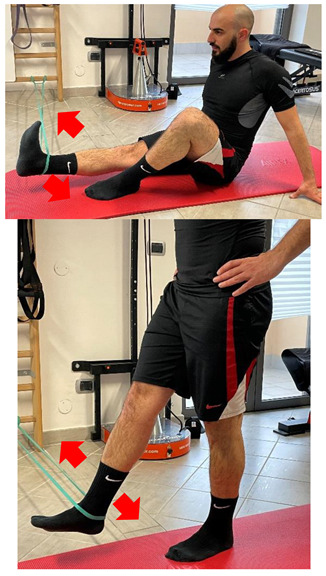	[23]
**Proprioceptive, neuromuscular, and stretching exercises**		
	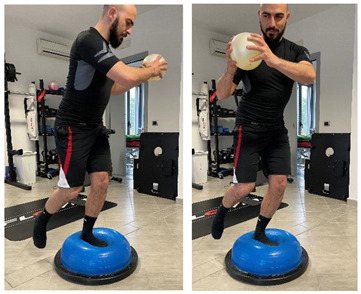	[23]
**Secondary prevention exercises**		
Eccentric side lunge stretch	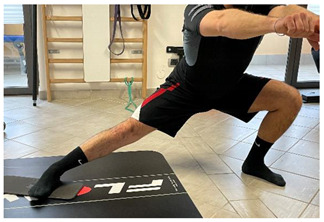 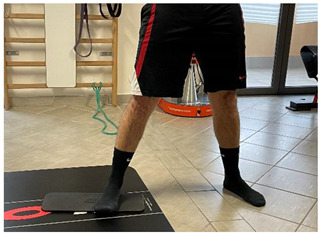	[98]
Copenhagen adductor prevention programs	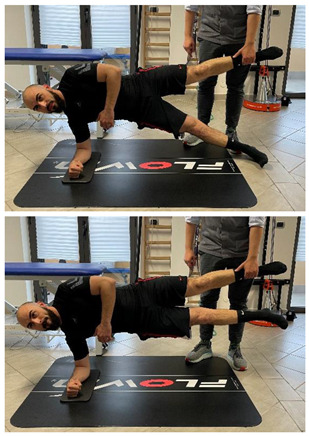	[99]

**Soleus-gastrocnemius**

**Table 5:** Soleus-gastrocnemius rehabilitation exercises.

Name	Image	Reference
**Isometric exercises**		
Isometric contraction with manual resistance	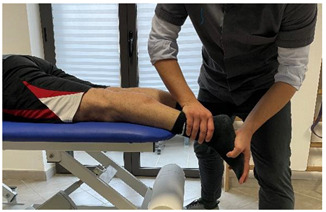 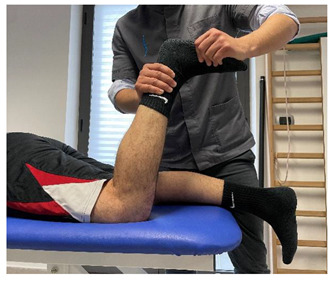	[23]
**Dynamic exercises**		
Concentric/eccentric contraction with manual resistance	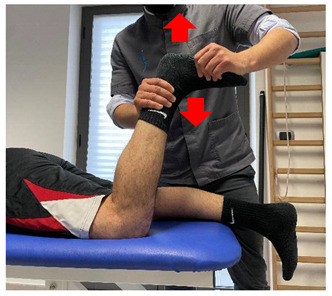	[23]
Concentric/eccentric heel raise	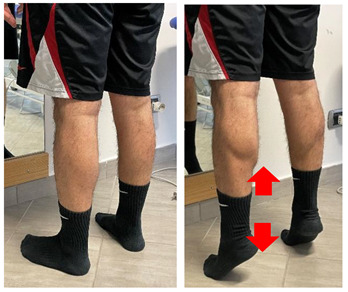	[23]
**Proprioceptive, neuromuscular, and stretching exercises**		
	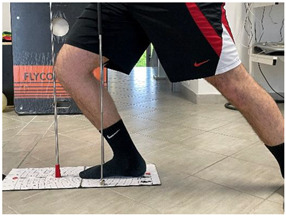	[23]

## 4. Return to Training (RTT) and Return to Play (RTP)

US examination upon complete RTT and a few days after the RTP is recommended [100]. There are no validated imaging criteria to guide the decision of a safe RTP. To date, no study has suggested US to guide the RTP decision, but a few studies have focused on MRI following hamstring injury [101,102,103,104]. Normalization of increased signal intensities on MRI is therefore not required for a successful RTP, since the signal alterations also persist at different weeks after the clinical healing of the injury, suggesting that functional recovery advances structural recovery at imaging [9].

The RTT process should be as individualized as possible, to allow a safe and fast return after a muscle injury. Regarding this point, the Italian consensus conference gave useful advice [101]. General assessment about this process is made up of some key points: absence of clinical symptoms [105,106,107]; absence of pain or tenderness during muscle palpation [10,107,108,109]; absence of pain on passive and active stretching [110]; absence of pain on isometric, concentric and eccentric contraction [10]; completion of the prescribed rehabilitation program [108]; MRI and US imaging [111,112]; subjective feelings of the player taken into account [113,114,115].

It is recommended that the athlete accomplishes a normal of week training of at least four sessions without pain, discomfort, or ‘fear’. During this week, performance can be monitored for normality by global positioning system (GPS) and heart rate data [116]; this performance control should be extended to competitions after RTP. The reference value, below which the positive judgement for RTP is postponed, is arbitrarily set at a maximum difference of 10% between preinjury data and the data recorded during the acquisition period following RTT. Furthermore, an evaluation of aerobic capacity is recommended. A VO2 max equal to at least 90% of their preinjury level seems to offer more guarantees for a safer RTP [101].

To define a set of tests to determine the correct timing of RTT is a difficult task. Specific assessment for each muscle group, laboratory tests aimed to assess muscle strength, and functional field tests could be adopted as criteria to define a safe RTT.

Based on the available literature, a list of tests has been defined to each muscle groups and are reported in Table 6.

The aim of these tests and their specific execution are out of the scope of the present paper, but the rationale behind each test is reported in the references and could be used to guide the RTT and RTP processes.

## 5. Conclusions

The present paper offers an overview of advice and recommendations on how to set up the rehabilitation process after indirect muscle injuries according to current evidence. The section on specific exercises for the most affected muscles groups adds a practical guide for the practitioner to apply the concepts reported in the guidelines to a real-life scenario. Only with the constant synergistic work between the various professionals involved, who work according to the highest scientific evidence available, the injured athlete can reach the maximum result of the rehabilitation process and return to their sport quickly and safety.

## Figures and Tables

**Table 1:** Indirect structural muscle injuries classification (adapted from [9]).

Severity	Site	Tissue	Relapse
3A: minor partial lesion	P: proximal	MF: myofascial	R0: first lesion
3B: moderate partial lesion	M: medium	MT: muscular belly and myotendinous junction	R1: first relapse
4: subtotal or total lesion and tendon avulsion	D: distal	T: central tendon or free	R2: second relapse
			R3: third relapse

**Table 6:** Return to play (RTP) specific tests for muscle groups.

**Hamstring**	
*Specific assessment*	Passive straight leg raise test [110,117,118]; Dynamic flexibility H test [119]
*Laboratory test*	Dynamometric tests (isometric, isotonic and isokinetic tests) [104,120,121]
*Field test*	Illinois Agility Test [101,122,123] Braking test [101] Backward running [124,125]
**Quadriceps**	
*Specific assessment*	Passive quadriceps stretch test [110,126]
*Laboratory test*	Dynamometric tests [107,120,121] Synchro plates test [121]
*Field test*	Illinois Agility Test [101,122,123] Braking test [101] Kicking test [10]
**Adductors**	
*Specific assessment*	Pubic stress test [127] Resisted hip adduction test [120,128] Squeeze test [129,130,131,132] Adductor passive stretching test [133]
*Laboratory test*	Adductor muscles strength assessed by dynamometric tests [104,120,121]
*Field test*	Kicking test [10] Carioca test [134,135]
**Soleus-gastrocnemius**	
*Specific assessment*	Heel-raise test [38,136,137] Ankle flexibility test [138,139,140]
*Laboratory test*	Dynamometric tests [107,119,120] Synchro plates test [121] Drop jump test [141,142,143]
*Field test*	Illinois Agility Test [101,142,143]

## Data Availability

Not applicable.
